# A cyclic azapeptide ligand of the scavenger receptor CD36/SR-B2 reduces the atherosclerotic lesion progression and enhances plaque stability in apolipoprotein E-deficient mice

**DOI:** 10.3389/fphar.2023.1204905

**Published:** 2023-05-30

**Authors:** Jade Gauvin, Geneviève Frégeau, Hanan Elimam, Liliane Ménard, David Huynh, Catherine Lê, Ahsanullah Ahsanullah, William D. Lubell, Huy Ong, Sylvie Marleau

**Affiliations:** ^1^ Faculty of Pharmacy, Université de Montréal, Montréal, QC, Canada; ^2^ Department of Biochemistry, Faculty of Pharmacy, University of Sadat City, Sadat City, Egypt; ^3^ Department of Biochemistry, Faculty of Pharmacy, Sinai University-Kantara Branch, Ismailia, Egypt; ^4^ Department of Chemistry, Université de Montréal, Montréal, QC, Canada; ^5^ Department of Chemistry, Quaid-i-Azam University, Islamabad, Pakistan

**Keywords:** atherosclerosis, CD36, azapeptide, lesion stability, efferocytosis, macrophages, necrosis

## Abstract

Atherosclerosis is a chronic inflammatory disease of the arterial walls that develops at predisposed sites. As a major risk factor for adverse cardiovascular pathology, atherosclerosis can progress to myocardial infarction and stroke, due to the rupture of unstable atherosclerotic lesions. Macrophage uptake of modified lipoproteins and metabolic dysfunction contributes significantly to the initiation and development of atherosclerotic lesions. The cluster of differentiation 36 receptor [CD36 (SR-B2)] plays a key role in atherosclerotic lesion progression and acts as an efferocytic molecule in the resolution of advanced plaque. In previous studies, linear azapeptide CD36 ligands were shown to exhibit anti-atherosclerotic properties. In the present study, a novel potent and selective macrocyclic azapeptide CD36 ligand, MPE-298, has proven effective in protecting against atherosclerosis progression. Features of greater plaque stability were observed after 8 weeks of daily injections with the cyclic azapeptide in apolipoprotein E-deficient mice fed a high-fat high-cholesterol diet.

## Introduction

Cardiovascular diseases (CVDs) account for the highest number of non-communicable disease deaths and premature deaths globally (Khan et al., 2020; Roth et al., 2020). In spite of advances in preventive measures and hypolipidemic therapy, the number of patients suffering from CVDs has nearly doubled over the last 30 years (Roth et al., 2020). The most prevalent manifestation of CVDs is ischemic heart disease (IHD), which is commonly caused by atherosclerosis, a chronic fibrofatty and inflammatory disease of the artery wall (Libby et al., 2019). Inflammation during atherosclerosis is a major factor in lesion initiation, progression, and, at advanced stages, plaque disruption (Libby et al., 2002). In the initiation and progression of atherosclerosis, blood monocytes and tissue macrophages contribute significantly. Macrophage uptake of modified lipoproteins can lead to conversion into inflammatory foam cells, which undergoe apoptosis and secondary necrosis in advanced stages of the disease (Wilson, 2022). Efferocytosis, the uptake of apoptotic cells by phagocytes such as macrophages, acts as a compensatory mechanism to reduce cell necrosis and lipid necrotic core formation in atherosclerotic lesions (Ge et al., 2022). The cluster of differentiation 36 receptor (CD36), a class B type 2 scavenger receptor family (SR-B2) membrane glycoprotein, is widely expressed in various mammalian cell types, including monocytes and macrophages. Playing major roles in long-chain fatty-acid transport, CD36 also regulates the uptake of oxidatively modified low-density lipoproteins (oxLDLs) (Febbraio et al., 2001; Febbraio and Silverstein, 2007). On the surfaces of monocytes and macrophages, CD36 mediates the internalization and metabolism of oxLDLs, leading to foam cell formation, vascular inflammation, and lesion progression (Febbraio and Silverstein, 2007). On the contrary, CD36 functions together with other cell surface proteins in efferocytosis to mitigate inflammation and to promote the resolution of atherosclerosis (Kourtzelis et al., 2020; Ma et al., 2023).

Previously, CD36 ligands of the growth hormone-releasing peptide family (e.g., GHRP-6) have been shown to reduce atherosclerosis progression, dampen mononuclear cell recruitment to lesion areas, increase cellular cholesterol efflux, and reverse cholesterol transport (Marleau et al., 2005; Bujold et al., 2009; Harb et al., 2009; Bujold et al., 2013). Azapeptide analogs of GHRP-6, in which an amino amide is replaced by a semicarbazide, have demonstrated CD36 selectivity (Proulx et al., 2012; Proulx et al., 2020). Notably, [aza-Tyr^4^]- and [aza-(*N*,*N*-diallylaminobut-2-ynyl)Gly^4^]-GHRP-6 (MPE-001 and MPE-003) were proven to be effective in reducing lesion progression, diminishing pro-inflammatory macrophage polarization, and lowering plasma inflammatory cytokines in a mouse model of atherosclerosis (Frégeau et al., 2020). Cyclic azapeptides (e.g., MPE-298), which were prepared by A^3^-macrocyclization, were shown to display *in vitro* unprecedented CD36 binding affinity (IC_50_ of 0.1 µM) (Ohm et al., 2021), whereas linear azapeptides presented an IC_50_ value of ∼1 µM (Possi et al., 2017) and superior potency in reducing NO production and levels of major pro-inflammatory mediators (notably, CCL2 and IL-1) (Zhang et al., 2017). Potent metabolically stable modulators of CD36, i.e., cyclic azapeptides, favored an anti-inflammatory mononuclear phagocyte phenotype and enhanced the cholesterol efflux (Danelius et al., 2019; Ohm et al., 2021). The therapeutic potential for improving the lesion stability by targeting CD36 with the cyclic azapeptide MPE-298 has now been investigated in a preclinical mouse model of atherosclerosis featuring apolipoprotein E-deficient (apoE^−/−^) mice fed a high-fat high-cholesterol (HFHC) diet.

## Methods

### Azapeptides

Azapeptide analogs of GHRP-6, MPE-003 (Frégeau et al., 2020), and MPE-298 (Supplementary Figure S1) were synthesized and characterized, as described previously (Zhang et al., 2014; Ahsanullah et al., 2019), and reconstituted in sterile 0.9% NaCl before injection. MPE-003 was used as a positive control due to its well-characterized anti-atherosclerotic effect in apoE^−/−^ mice (Frégeau et al., 2020).

### Mice

The apoE^−/−^ mice were originally purchased from the Jackson Laboratory (Bay Harbor, ME, United States) and bred in-house by mating apoE^−/−^ female and male mice. The mice were housed in ventilated cages in a pathogen-free area before transferring them to a conventional environment for the duration of the protocol. Food and water were provided *ad libitum*. From 4 weeks of age, the male mice were fed a HFHC diet containing 40% of fat and 1.25% cholesterol (D12108, Research Diets, Inc., New Brunswick, NJ, United States).

### Experimental protocol

All experimental protocols were approved by the Institutional Animal Ethics Committee and performed in accordance with the guidelines for the care and use of laboratory animals provided by the Canadian Council on Animal Care and the US National Institute of Health. At 4 weeks of age, the male mice were randomly assigned to four experimental groups: group 1: no treatment (*n* = 8); group 2: 0.9% NaCl (*n* = 11); groups 3 and 4: 300 nmol/kg azapeptide, MPE-003 (*n* = 13), and MPE-298 (*n* = 11), respectively. At 12 weeks of age, group 1 was euthanized with an isoflurane overdose, followed by exsanguination, providing biochemical and histological samples that were used as baseline values and to establish the level of basal lesion development before initiating treatment. Groups 2–4 received daily subcutaneous (s.c.) injections (1 μL/g) from weeks 12 to 20, respectively, into the loose skin of the neck above the shoulder. At week 12 for group 1 and at week 20 for the other groups, blood was collected directly from the heart of the anesthetized mice. Glycemia was assessed with Accu-Chek strips from the blood drawn from the subclavian vein at 12 and 20 weeks for all groups. For analyses, plasma and tissues were snap frozen in dry ice and stored at −80°C. The aorta was dissected, the thoracic portion was fixed in 10% neutral buffered formalin, and the abdominal aorta with iliac arteries was snap frozen. Formalin-fixed tissues, except for the aorta, were processed for standard immunohistochemical procedures by the histology core of the *Institut de recherche en immunologie et en cancérologie* (Institute for Research in Immunology and Cancer, IRIC) of the Université de Montréal.

### Morphometric analyses of aortic lesions

Aortic atherosclerotic lesion areas were assessed by *en face* analysis after lipid staining with Oil Red O, as described previously (Marleau et al., 2005). Lesion-containing aortic crosses were assessed as the percentage of stained lesions relative to the total aortic cross-section area. The groups were comparable in terms of the aortic cross-surface area (Supplementary Figure S2). Cross sections of the aortic sinus were prepared by the IRIC histology core facility from hearts fixed in 10% neutral buffered formalin. The hearts were embedded in paraffin and sectioned along a plane parallel to the left atrium in sequential 4-μm sections starting from the three valve cusps of the aortic sinus. Sections were cut using a microtome and mounted on a microscope slide. The sections were stained with hematoxylin–eosin (H&E) (Gill Hematoxylin and intensified Eosin Y, Thermo Fisher Scientific, Mississauga, ON, Canada) and scanned using the NanoZoomer 2.0-HT scanner (Hamamatsu Photonics, Shizuoka, Japan) to provide digitalized images. The sinus plaque area was measured at 40-μm intervals and up to four sections per mouse, five mice per group. Atherosclerotic lesion areas were assessed as the percentage of the total aortic sinus area. Necrotic areas of aortic roots were determined as acellular regions of H&E-stained slices using Adobe Photoshop CS3 software (Adobe Systems Incorporated, San José, CA, United States) and expressed as the percentage of sinus areas, as described previously (Frégeau et al., 2020). Each section was analyzed by two blinded individuals.

### Plasma cholesterol and free fatty acids

The total plasma cholesterol was assayed using the Infinity™ total cholesterol reagent and calibrator (Thermo Fisher Scientific, Mississauga, ON, Canada). Free fatty acids (FFAs) were assessed, as previously described (Bessi et al., 2012).

### Cytokines

Plasma cytokines were assayed using mouse ELISA Ready-SET-Go!™ Kits (eBioscience, San Diego, CA, United States), according to the manufacturer’s instructions.

### Immunohistochemistry

Cross sections (4 μm) of the brachiocephalic artery (BCA) from five mice per group were cut at 40-μm intervals, processed, and stained for inducible nitric oxide synthase (iNOS or nitric oxide synthase 2, NOS2), CD206 (also known as the mannose receptor C type 1, MRC1), and caspase-3 by the IRIC histology core facility, as detailed previously (Frégeau et al., 2020). Primary and secondary antibodies are described in Supplementary Table S1. The isotype control antibody, in addition to negative control staining for nonspecific binding, has been assessed previously (Frégeau et al., 2020). Hematoxylin was used to counterstain the sections, and the images were acquired using a ×20 objective, the NanoZoomer 2.0-HT digital scanner, and NDP.view2 software (Hamamatsu Photonics, Shizuoka, Japan). The results were expressed as total positive cells in atherosclerotic lesions. Cell counts were assessed both manually using Adobe Photoshop CS3 software (San José, CA, United States) and using an automatic counter with ImageJ software (National Institutes of Health, Bethesda, MD, United States). Collagen in the BCA cross sections was stained with Masson’s trichrome and was measured as the percentage of collagen relative to the total lesion area using Adobe Photoshop CS3 software. Two to four sections per mouse per the five mice group were analyzed by two blinded individuals.

### Immunofluorescence

Immunofluorescence (IF) staining was performed on BCA cross sections by the IRIC histology core facility, as previously published (Frégeau et al., 2020). Briefly, antigen retrieval of de-paraffined slides was carried out by proteolytic-induced epitope retrieval (PIER), prior to the application of diluted primary antibodies against MerTK (1:50, mouse monoclonal IgG2b, Santa Cruz, Dallas, TX, United States) and CD36 (1:100, rabbit polyclonal IgG, Bioss, Woburn, MA, United States) for 1 h at room temperature. The corresponding secondary antibodies conjugated with Alexa Fluor^®^ were applied for 1 h at room temperature for each section to detect MerTK (1:200, AF555 goat anti-mouse IgG, Invitrogen, Waltham, MA, United States) and CD36 (1:200, AF488 goat anti-rabbit, Invitrogen, Waltham, MA, United States). Counterstaining of the sections was realized using the ProLong™ Gold Antifade Mountant (Invitrogen, Waltham, MA, United States) along with 4′6-diamindino-2-phenylindole (DAPI, 30 nM). Negative controls (omission of primary antibodies) were performed (Supplementary Figure S3). The slides were coverslipped manually and scanned at ×40 using the NanoZoomer 2.0-HT scanner (Hamamatsu Photonics, Shizuoka, Japan) at a fluorescence intensity setting of 5 for all channels (FITC/TxRed/DAPI) to provide high-resolution images of the sections. The images were analyzed using Adobe Photoshop CS3 software (Adobe Systems Inc., San José, CA, United States). Manders’ coefficients were determined using ImageJ software (National Institutes of Health, Bethesda, MD, United States).

### RT-qPCR analyses

Extraction of the total mRNA from abdominal aortas and iliac arteries was performed using the Ribozol™ RNA Extraction Reagent (VWR International, Radnor, PA, United States) with the PureLink™ RNA Micro Kit (Invitrogen, Waltham, MA, United States), as described previously (Frégeau et al., 2020). For the housekeeping gene, β-actin was used to normalize mRNA levels. The determination of the relative mRNA expression level of genes was carried out using the comparative CT (2^−ΔΔCt^) method. The protocol and primers are detailed in Supplementary Table S2.

### Statistical analyses

All data were analyzed using GraphPad Prism (v.9.5.1, San Diego, CA, United States) and expressed as mean ± SEM. Normally distributed data from independent groups were compared using one-way ANOVA, followed by Tukey’s *post hoc* test for multiple comparisons, and non-normally distributed data were compared using the Kruskal–Wallis test with Dunn’s *post hoc* test for multiple comparisons, unless stated otherwise. Statistical significance was considered at a *p*-value <0.05.

## Results

### Azapeptides reduce atherosclerotic lesion progression and cell death

**FIGURE 1:**
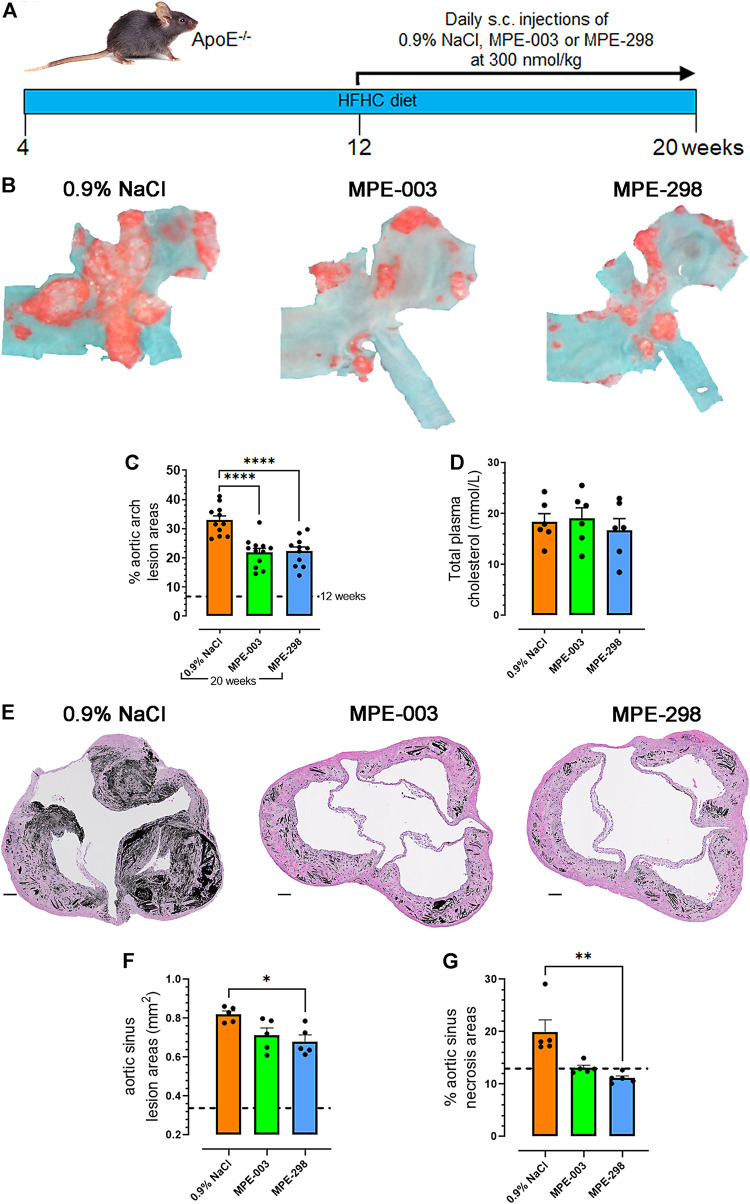
Azapeptides reduce atherosclerotic lesion progression and necrotic areas. **(A)** Study design. **(B)** Representative photomicrographs of aortic arches stained *en face* with Oil red O. **(C)** Percentage aortic arch lesion areas expressed as bar graphs and dot plots, where each dot represents the lesion area for each mouse (*n* = 11–12 mice per group). **(D)** Bar graphs and dot plots represent the mean total plasma cholesterol levels (mmol/L), in which each dot represents the value of a single mouse (*n* = 6 mice per group). **(E)** Representative photomicrographs of aortic sinuses after staining with hematoxylin–eosin (scale bar: 100 μm). Necrotic areas are indicated in black. **(F)** Bar graphs and dot plots represent the mean sinus lesion areas (mm^2^), in which each dot is the mean lesion area of 3–4 cross sections per mouse (*n* = 5 mice per group). **(G)** Bar graphs and dot plots represent the mean aortic sinus necrotic areas (%), in which each dot is the mean necrotic area of 3–4 cross sections per mouse (*n* = 5 mice per group). Data are mean ± SEM. *p < 0.05, **p < 0.01, ***p < 0.001, and ****p < 0.0001, as assessed by one-way ANOVA and Tukey’s *post hoc* test.

Prior to euthanasia at week 20, apoE^−/−^ mice were fed a HFHC diet starting from 4 weeks of age (Figure 1A). At 12 weeks of age, a group was euthanized to determine lesion areas before initiating treatment. Vehicle (0.9% NaCl), cyclic azapeptide MPE-298 (300 nmol/kg), and linear azapeptide MPE-003 positive control (300 nmol/kg), all were administered daily by s.c. injections for 8 weeks (Frégeau et al., 2020). Reduced lesion areas were revealed in animals treated with azapeptides compared to those exposed to the vehicle as shown in photomicrographs of Oil Red O-stained aortic arches from apoE^−/−^ mice (representative images in the top row, Figure 1B). The lesion areas grew swiftly from 7% to 33% as vehicle-treated mice aged from 12 to 20 weeks old (dotted line on Figure 1C). Linear and cyclic azapeptides MPE-003 and MPE-298 reduced aortic arch lesion areas by 33% (*p* < 0.0001; from 32.9% ± 1.5% to 21.9% ± 1.4%) and 32% (*p* < 0.0001; from 32.9% ± 1.5% to 22.2% ± 1.5%), respectively. Vehicle- and azapeptide-treated mice exhibited no significant differences in elevated cholesterol levels (Figure 1D). Photomicrographs of aortic sinuses (Figure 1E and Supplementary Figure S4) demonstrated that MPE-298 caused a modest 17% (*p* < 0.05) reduction of lesion areas (Figure 1F) and significantly reduced necrosis by 44% (*p* < 0.01) compared to that of vehicle-treated mice (Figure 1G). A less pronounced trend was observed in the mice treated with MPE-003 (Figures 1E–G). Azapeptide treatment had no observable effect on the body weight nor food intake (Supplementary Figures S5A, B). In addition, no change in glycemia nor free fatty acid levels was observed in azapeptide-treated mice fed a HFHC diet at 20 weeks of age, compared to that of vehicle-treated mice. (Supplementary Figures S6A, B).

### Azapeptides reduce cell apoptosis and promote plaque stability in brachiocephalic arteries

**FIGURE 2:**
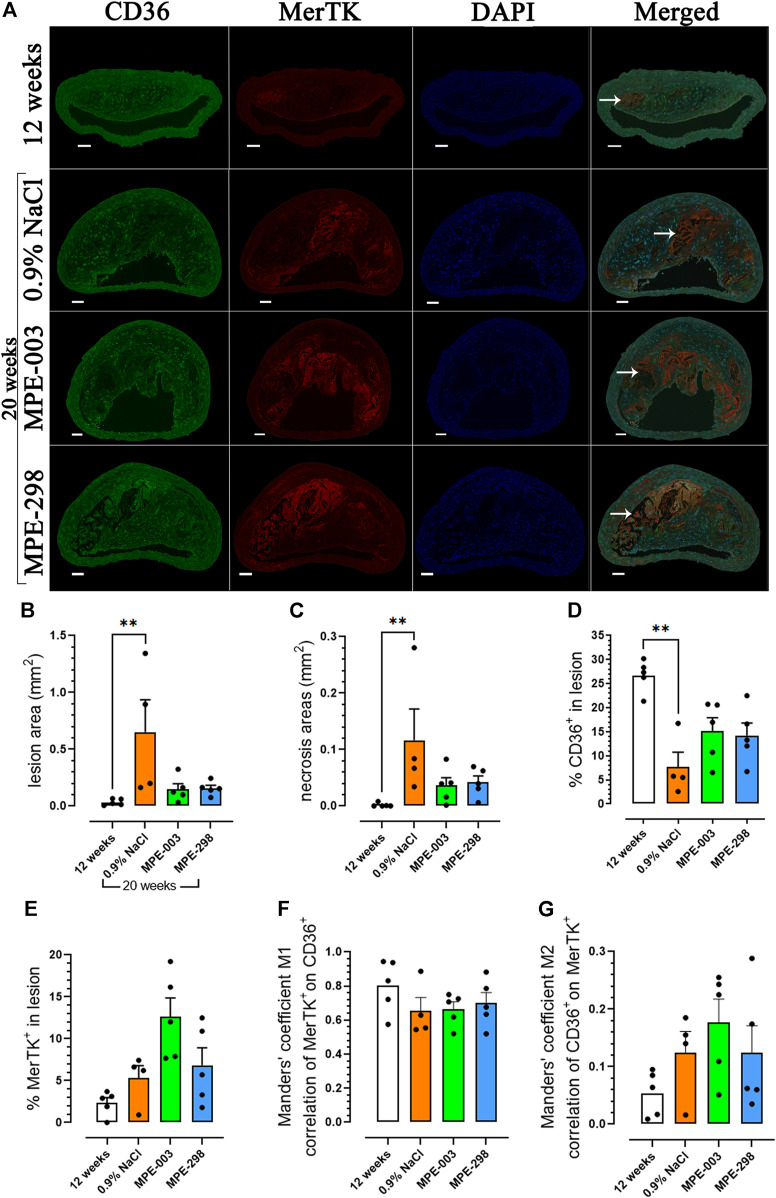
MerTK and CD36 are colocalized in the BCA. **(A)** Immunofluorescence staining of CD36 (green), MerTK (red), DAPI (blue), and their colocalization in the BCA (scale bar: 50 μm). Arrows indicate the necrotic core. **(B)** Bar graphs and dot plots represent the mean lesion areas (mm^2^) and **(C)** necrotic areas (mm^2^) in the BCA. Bar graphs and dot plots represent the mean immunofluorescence staining of **(D)** CD36 (%) and **(E)** MerTK (%) in the lesion area. **(F)** Bar graphs and dot plots of the Manders’ coefficient M1 (red on green). **(G)** Bar graphs and dot plots of the Manders’ coefficient M2 (green on red). Data are mean ± SEM. Each dot is the mean of 3–4 cross sections per mouse (*n* = 4–5 mice per group). *p < 0.05 and **p < 0.01, as assessed by the Kruskal–Wallis test and Dunn’s *post hoc* test.

Immunofluorescence staining of the merged CD36, MerTK, and DAPI photomicrographs (Figure 2A) was used to determine the lesion size and necrotic areas of BCAs. In the lesion, CD36 immunofluorescence staining was widely distributed and MerTK staining was concentrated mainly at the periphery of the necrotic core. Lesion and necrotic areas were elevated over 20-fold in 20-week-old compared to that in 12-week-old apoE^−/−^ mice (Figures 2B, C). In 20-week-old apoE^−/−^ mice fed a HFHC diet, staining indicated a ∼four-fold decrease in CD36 compared to that in 12-week-old mice. On the contrary, animals treated with azapeptides tended to exhibit increased CD36 staining in lesion areas compared to that in the vehicle-treated mice (Figure 2D). In contrast, MerTK staining tended to be higher in the MPE-003-treated mice than that in vehicle-treated mice (Figure 2E). A high level of colocalization of MerTK to CD36 immunofluorescence staining was indicated by the Manders’ coefficient M1 (Figure 2F), but CD36 was more largely distributed with a lower degree of colocalization with MerTK according to the Manders’ coefficient M2 (Figure 2G). MerTK-immunostained areas correlated with necrotic areas (Supplementary Figure S7).

**FIGURE 3:**
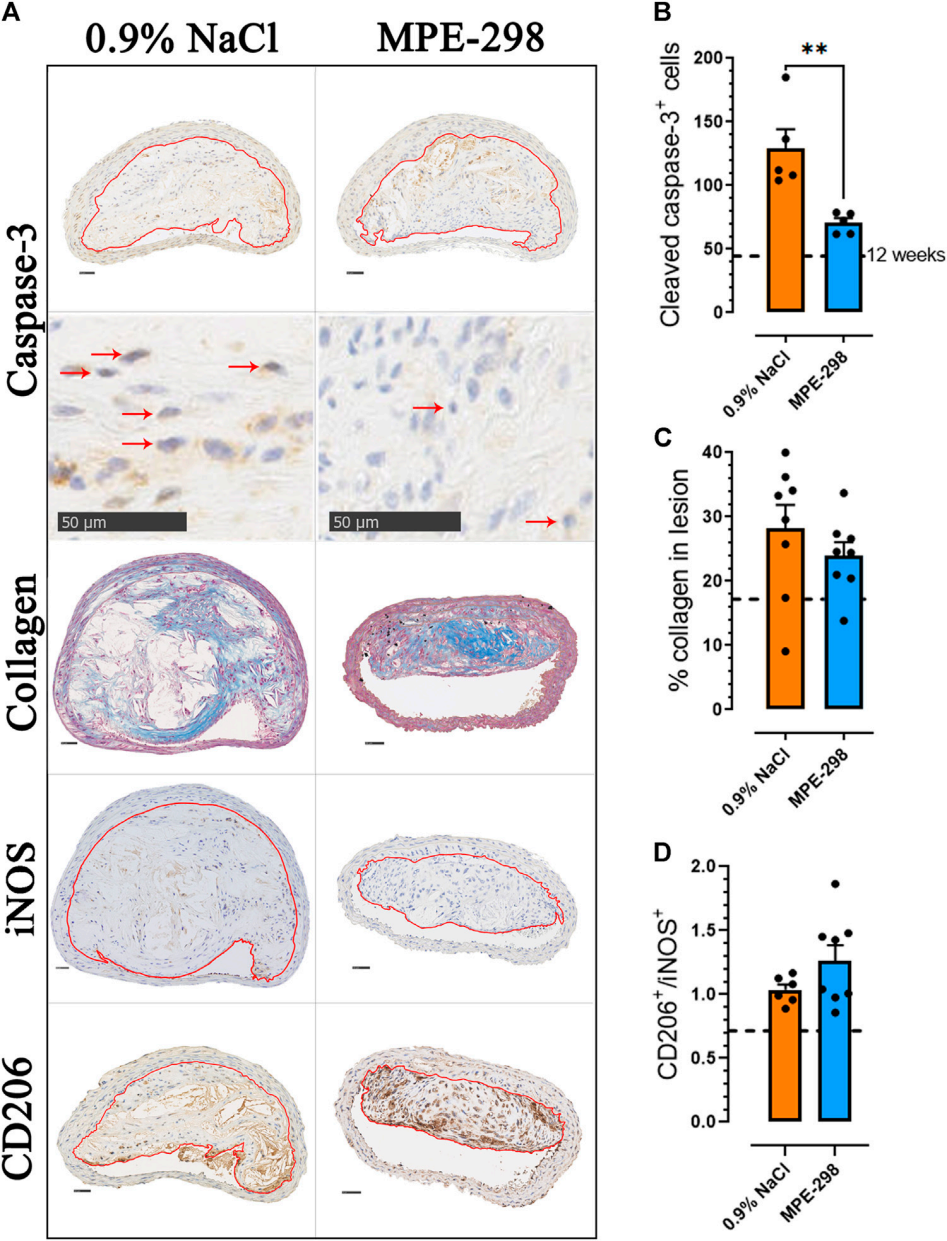
Azapeptides promote plaque stability in the BCA. **(A)** Representative cross sections of the stained BCA for caspase-3, collagen, iNOS, and CD206 (scale bar: 50 μm), with an enlarged area for caspase-3 immunostaining. Arrows indicate caspase-3-positive cells. **(B)** Bar graphs and dot plots of the mean caspase-3^+^ cell count in the BCA lesion area (*n* = 5 mice per group). **(C)** Bar graphs and dot plots represent the mean collagen expressed as the percentage of the lesion area (*n* = 8 mice per group). **(D)** Bar graphs and dot plots of the CD206-to-iNOS-positive cell ratio in the BCA lesion area (*n* = 6–8 mice per group). Data are mean ± SEM. Each dot is the mean of 3–4 cross sections per mouse. *p < 0.05 and **p < 0.01, as assessed by the Mann–Whitney U test for **(B)** and unpaired *t*-tests for **(C)** and **(D)**.

In addition to reduced necrosis, apoptosis in BCA lesions (Figure 3A) was evaluated by assessing the cleaved caspase*-*3 immunoexpression (Figure 3B); active caspase-7 or c-PARP antibodies might have been used to support the results (Bressenot et al., 2009). In mice treated with MPE-298, immunostained caspase-3 was reduced by 45% (*p* < 0.01) compared to that in vehicle-treated animals (Figure 3B). It is generally well-recognized that apoptosis correlates with the lesion stage and plaque vulnerability (Van Vre et al., 2012) and that an imbalance in efferocytosis may lead to secondary necrosis. Our results show that both apoptosis and necrosis appeared to be reduced in the MPE-treated mice. BCA cross sections were further analyzed for collagen deposition by evaluating Masson’s trichrome-stained areas. As indicated in representative photomicrographs of BCA lesions from vehicle- and MPE-298-treated apoE^−/−^ mice (Figure 3A), collagen-stained areas did not reveal significant differences between the two groups (Figure 3C). Markers of macrophage inflammatory and non-inflammatory phenotypes were assessed on immunostained photomicrographs of CD206^+^ and iNOS^+^ cells in BCA lesions (Figure 3A), and the CD206/iNOS-positive cell ratio was increased by 22% but did not reach statistical significance (Figure 3D).

### MPE-298 reduces biomarkers of plaque instability and systemic inflammation

**FIGURE 4:**
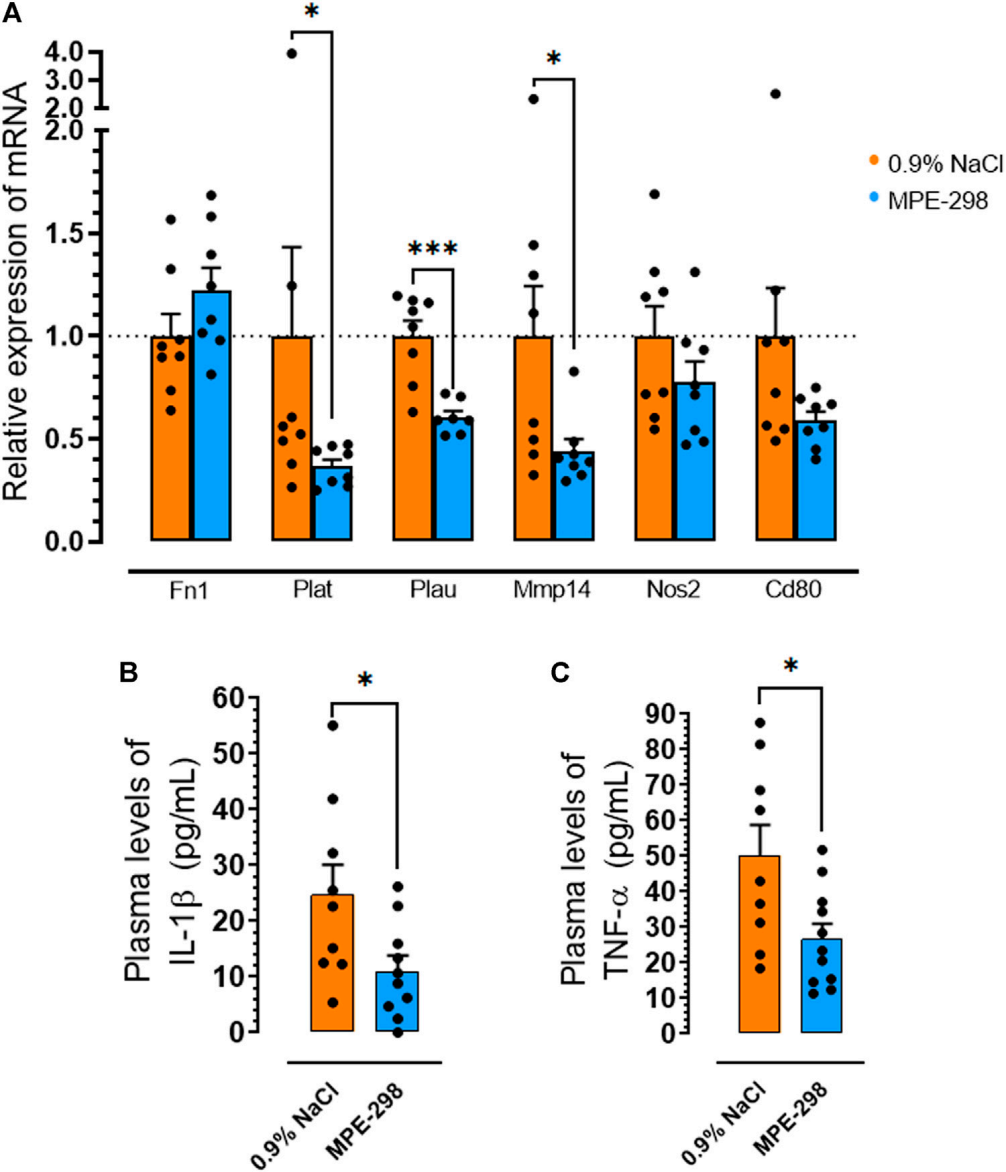
MPE-298 enhances plaque stability and reduces systemic inflammation. **(A)** Bar graphs and dot plots of the abdominal and iliac arterial tissue relative to the mRNA levels of fibronectin (Fn1), tissue-plasminogen activator (Plat), urokinase-type plasminogen activator (Plau), matrix metalloproteinase-14 (Mmp14), nitric oxide synthase 2 (Nos2), and Cd80 (*n* = 8 mice per group). Bar graphs and dot plots of plasma **(B)** IL-1β and **(C)** TNF-α levels (*n* = 9–11 mice per group). Data are mean ± SEM. *p < 0.05, **p < 0.01, and ***p < 0.001, as assessed by the Mann–Whitney U test for **(A)** and unpaired *t*-tests for **(B)** and **(C)**.

Abdominal aortas and iliac arteries were collected and extracted for selected gene quantification. These areas were second in terms of lesion burden after the aortic arch (Marleau et al., 2005; Harb et al., 2009; Frégeau et al., 2020). Additional markers of plaque instability, including tissue- and urokinase-type plasminogen activators genes (Plat and Plau) and metalloproteinase-14 (Mmp14), were assessed by qPCR of abdominal aortas. In mice treated with cyclic azapeptide MPE-298, mRNA levels of Plat, Plau, and Mmp14 were reduced by 63% (*p* < 0.05), 44% (*p* < 0.001), and 56% (*p* < 0.05), respectively, compared to those in vehicle-treated animals (Figure 4A). Moreover, pro-inflammatory cytokine plasma levels of IL-1β (Figure 4B) and TNF-α (Figure 4C) were shown to be reduced by 55% (*p* < 0.05) and 47% (*p* < 0.05), respectively, using ELISA assays in the MPE-298-treated mice compared to those in the animals exposed to the vehicle.

## Discussion

Cyclic azapeptide MPE-298, a potent and selective CD36 ligand (Zhang et al., 2017; Ohm et al., 2021), exhibited anti-atherosclerotic properties similar to those of linear azapeptide MPE-003, for which vasculo-protective effects in hypercholesterolemic apoE^−/−^ mice were previously reported (Frégeau et al., 2020). Although both azapeptides elicited a reduction of about 33% of lesion areas in the aortic arch in 20-week-old apoE^−/−^ mice (Figures 1B,C), azapeptide MPE-298 induced a striking reduction of necrotic areas in the aortic sinus (Figures 1E,G). The BCA is a site of predilection for the development of unstable lesions in apoE^−/−^ mice (Williams et al., 2002). Sequential slices of the BCA were analyzed by immunofluorescence staining for the presence of the receptors MerTK and CD36, which are implicated in efferocytosis and markers of necrosis and apoptosis, collagen, and macrophage phenotypes. Although collagen degradation may render atherosclerotic plaques vulnerable to rupture (Rekhter, 1999), azapeptide MPE-298 caused no change in collagen accumulation or degradation in BCA lesions, as assessed by Masson’s trichrome stain histology (Figure 3C).

Macrophages, respectively, play critical roles in atherosclerotic lesion progression and resolution by mediating the uptake and metabolism of oxidized lipoproteins, by unloading a cholesterol surplus, and by clearing apoptotic cells through efferocytosis (Doran et al., 2020). Among receptors implicated in efferocytosis, MerTK has been characterized as the main macrophage receptor for apoptotic cell phagocytosis (Garbin et al., 2013). The scavenger receptor CD36 also contributes to the apoptotic cell uptake (Febbraio and Silverstein, 2007). The loss of MerTK and CD36 as phagocyte receptors has been shown to result in the inability to remove apoptotic cells (Wang et al., 2020). An impaired capacity to perform apoptotic cell clearance in atherosclerosis leads to secondary apoptotic cell necrosis, thus promoting inflammation and plaque instability (Yurdagul et al., 2017). Unstable plaques have been characterized to have a large necrotic core rich in cellular debris and extracellular lipids, bordered by a ring of phagocytic cells (van der Wal and Becker, 1999). In agreement, immunofluorescence staining indicated MerTK localization, particularly at the periphery of the necrotic core, associated with colocalized CD36. A broad distribution of CD36 immunofluorescence was observed over the lesion area (Figure 2A). Lesion and necrotic areas, both increased with age from 12 to 20 weeks in vehicle-treated apoE^−/−^ mice but tended to decrease upon treatment with azapeptides (Figures 2B, C). In vehicle-treated apoE^−/−^ mice, CD36 immunofluorescence in the BCA lesion decreased with age from 12 to 20 weeks of age, possibly as consequences of elevated cell apoptosis (Figure 3B) and necrosis. On the contrary, CD36 immunofluorescence tended to increase upon treatment with azapeptides (Figure 2D). Compared to vehicle-treated mice, animals treated with azapeptides exhibited a similar tendency for increased MerTK immunofluorescence staining, which was higher for MPE-003 (Figure 2E). A limitation to these studies is due to the inability to distinguish between intact cell surface transmembrane receptors and shed-receptor forms (sMER) by the anti-MerTK antibody used in the immunofluorescence assay. The sMER form has been reported to lower efferocytosis efficiency in opposition to the effects of MerTK (Thorp et al., 2011). The release of soluble CD36 into lesion areas could also not be assessed. The phenotypic heterogeneity of macrophages largely depends on the environment and inflammatory mediators. Macrophages, which exhibit an increase in the expression of the nuclear receptor peroxisome proliferator-activated receptor (PPAR)γ and liver X receptor (LXR)α, display enhanced efferocytosis, have the ability to resolve inflammation (Korns et al., 2011), and take on the resolution-prone M2 macrophage phenotype (Zhong et al., 2018; Wang et al., 2020). In agreement, a trend for the increased M2-to-M1 ratio was observed in MPE-298-treated mice (Figure 3D). The linear azapeptide MPE-001 has been previously shown to stimulate the PPARγ–LXRα pathway (Mellal et al., 2019) and to reduce pro-inflammatory macrophages (Frégeau et al., 2020). The GHRP-6 peptide analog EP-80317, which serves as a ligand of CD36, has also been shown to increase the protein levels of ATP-binding cassette (ABC) transporters and the macrophage cholesterol efflux (Bujold et al., 2009). Reduced systemic inflammation was exhibited in mice treated with MPE-298, which diminished plasma TNF-α and IL-1β levels. As shown previously, the second site of predilection of atherosclerotic lesion areas in our model is the abdominal aorta and iliac arteries (Marleau et al., 2005; Harb et al., 2009; Frégeau et al., 2020). In abdominal aorta of mice treated with MPE-298, mRNA levels of the membrane type metalloproteinase Mmp14, which plays a role in plaque rupture (Ray et al., 2004), were reduced (Figure 4B), as previously observed on treatment with linear azapeptides (Frégeau et al., 2020). Considering that IL-1β, TNF-α, and oxidized LDL all upregulate Mmp14 in macrophages (Ray et al., 2004), the effect of MPE-298 in lowering cytokine levels was in accordance with the reduction of metalloproteinase mRNA levels. Along with the reduction of mRNA levels of Mmp14, those of serine proteinases Plat and Plau, as markers of instability, were significantly decreased in the abdominal aorta of mice treated with MPE-298 (Figure 4A). The determination of protein levels should confirm these observations in future works. The latter members of the fibrinolytic cascade have been identified in smooth muscle cells and macrophages along the margin of the necrotic core in human atherosclerotic lesions (Dollery and Libby, 2006). In addition to their fibrinolytic properties, tissue- and urokinase-type plasminogen activators also interfere with efferocytosis, by competing for receptors of eat-me signals, such as Gas6 in apoptotic cells (Yang et al., 2010). The macrophage-targeted Plau overexpression has been shown to be deleterious in an apoE^−/−^ mouse model of atherosclerosis (Hu et al., 2015). An elevated Plat expression has been reported in advanced atherosclerotic apoE^−/−^ plaques (Zhi et al., 2013). On the contrary, mRNA levels of fibronectin (Fn1), a substrate of Plau and Plat, were not modulated in mice treated with the cyclic azapeptide MPE-298 (Figure 4A). One limitation to the present study is the use of only male mice, because increased risk of atherosclerosis is gender-dependent. The cardiovascular effect of azapeptides on both sexes merits further investigation.

Despite a short terminal half-life of 20 min, MPE-298 exerted atheroprotective effects after long-term daily s.c. injections. The short plasma half-life of the azapeptide did not correlate with efficacy. Considering the presumed protease-stability of MPE-298, further investigation of azapeptide internalization, intracellular disposition in the macrophages, and signaling pathways, all are currently being investigated in our laboratory.

In conclusion, daily treatments with the CD36 ligand cyclic azapeptide MPE-298 reduced lesion progression in apoE^−/−^ mice fed a HFHC diet from 4 weeks of age, in the same manner of the linear azapeptide MPE-003. Moreover, the cyclic azapeptide MPE-298 promoted plaque stability in the BCA, aortic sinus, and most likely the abdominal aorta. Additional investigations will be required to determine whether improved plaque stability associated with MPE-298 treatment can be observed in other regions of the arterial tree. Treatment with cyclic azapeptide MPE-298 tended to increase CD36 immunofluorescence and induced a 45% (*p* < 0.01) reduction in immunostained caspase-3 as a marker of apoptotic cells in BCA lesions. Consistent with diminished apoptotic cell amounts, plasma TNF-α and IL-1β cytokine levels were decreased in the azapeptide-treated animals. Furthermore, immunofluorescence staining indicated that treatment with azapeptide increased MerTK at the periphery of the necrotic core that colocalized with CD36 in the BCA. Considering the roles of MerTK and CD36 as phagocytic receptors, azapeptides (e.g., MPE-298) may promote macrophage efferocytosis, in addition to attenuating inflammation and enhancing plaque stability. Our results support the development of cyclic azapeptides as CD36 ligands, with potential plaque-stabilizing properties against atherosclerosis progression.

## Data Availability

The original contributions presented in the study are included in the article/Supplementary Material; further inquiries can be directed to the corresponding author.
