# Supplementary material for: A cyclic azapeptide ligand of the scavenger receptor CD36/SR-B2 reduces the atherosclerotic lesion progression and enhances plaque stability in apolipoprotein E-deficient mice
Source: Front Pharmacol. 2023 May 30;14:1204905. doi: 10.3389/fphar.2023.1204905 (PMC10270736; doi:10.3389/fphar.2023.1204905)
Supplement: Supplementary file 1 [file DataSheet1.PDF]

Supplementary Material

A cyclic azapeptide ligand of the scavenger receptor CD36/SR-B2 reduces atherosclerotic lesion progression and enhances plaque stability in apolipoprotein E-deficient mice

Jade Gauvin, Geneviève Frégeau, Hanan Elimam, Liliane Ménard, David Huynh, Catherine Lê, Ahsanullah, William D. Lubell, Huy Ong, Sylvie Marleau*

*** Correspondence:** Corresponding Author: sylvie.marleau@umontreal.ca

1 Supplementary Figures and Tables

1.1 Supplementary Tables

Supplementary Table 1. Immunohistochemistry antibodies

Marker	Primary antibody	Dilution	Source	Secondary antibody	Dilution	Source
CD206	Goat polyclonal IgG	1:50	R&D Systems, Minneapolis, MN, USA	Corresponding biotin-conjugated antibody	1:100	Jackson Immuno Research, West Grove, PA, USA
iNOS	Rabbit polyclonal IgG	1:2000	Novus Biological, Centennial, CO, USA			
Caspase-3	Rabbit polyclonal IgG	1:100	Biocare Medical, Pacheco, CA, USA			

Supplementary Table 2. qPCR murine primer sequences

Gene	Primer	Product length (bp)	NCBI Gene ID
β-actin	Forward CAGCAAGCAGGAGTACGATGA<br>Reverse GAAAGGGTGTAAAACGCAGCTC	93	11461
Cd80	Forward TTCAGACCGGGGCACATACAGC<br>Reverse TGTTGGGGGTAGAGAAGTCAGCTTTG	116	12519
Fibronectin (Fn1)	Forward TGCAGACCTACCCAGGCACAACT<br>Reverse TGGATGGGGTGGGAATTGGGCT	82	14268
Nos2	Forward ACCTTGGTGAAGGGACTGAGCTGT<br>Reverse TCCAACGTTCTCCGTTCTCTTGCAGT	112	18126
Mmp14	Forward GCCCTCTGTCCCAGATAAGCCCAA<br>Reverse CCAGAACCATCGCTCCTTGAAGACAA	121	17387
Plat	Forward AAAGAAGCAAGCACTCTCGGGACACA<br>Reverse GGTCAGGCAATGGGAAAGCCAGTC	105	18791
Plau	Forward GCTCTGTCTGTCATCCATCCAGTCCT<br>Reverse GGGCTCTAGACAGCAGTTCGGTGA	80	18792

1.2 Supplementary Figures

(A)

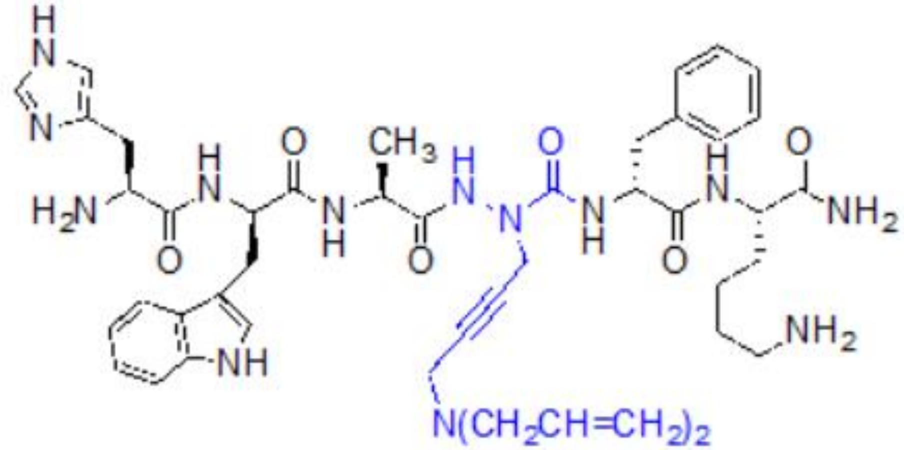

MPE-003

His-D-Trp-Ala-[aza-(N,N-diallylaminobut-2-ynyl)Gly^{4}]-D-Phe-Lys-NH_2

(B)

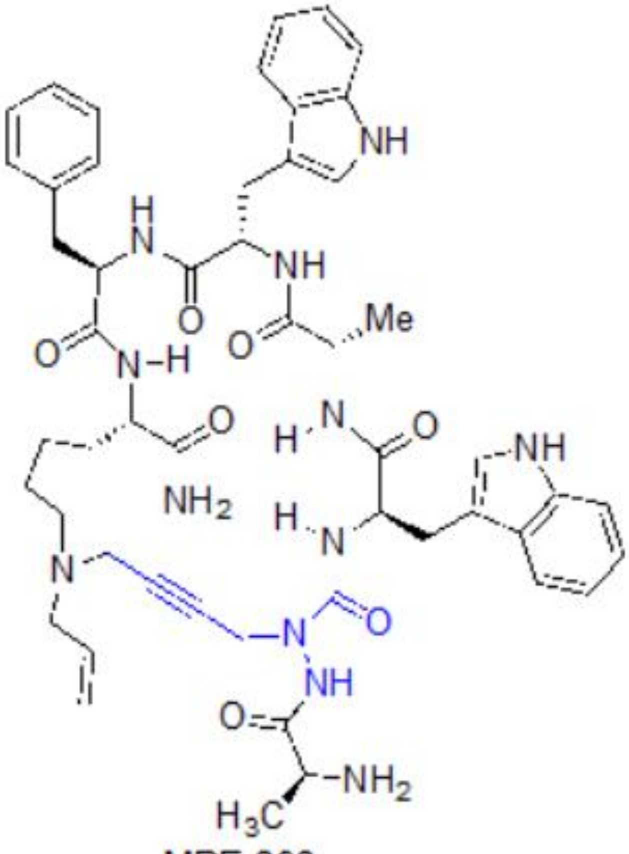

MPE-298

Ala-AzapropargylGly-D-Trp-Ala-Trp-D-Phe-Lys(allyl)-NH_2

Supplementary Figure 1. Molecular structure of azapeptides (A) MPE-003 and (B) MPE-298.

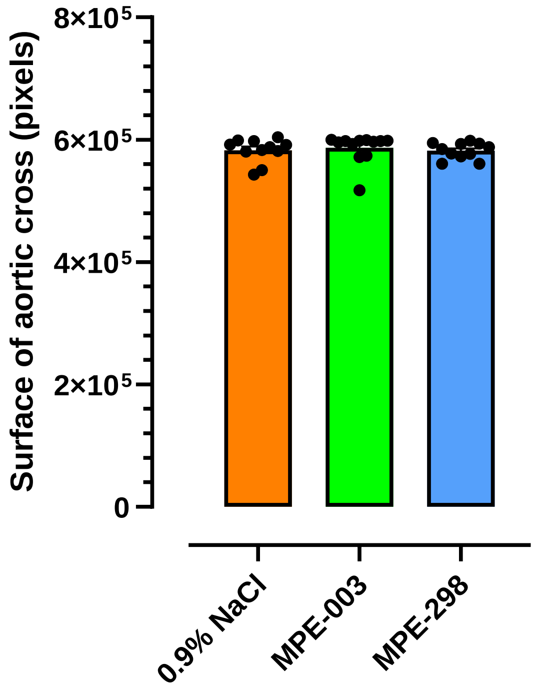

Supplementary Figure 2. All groups have comparable aortic cross pixel surface.

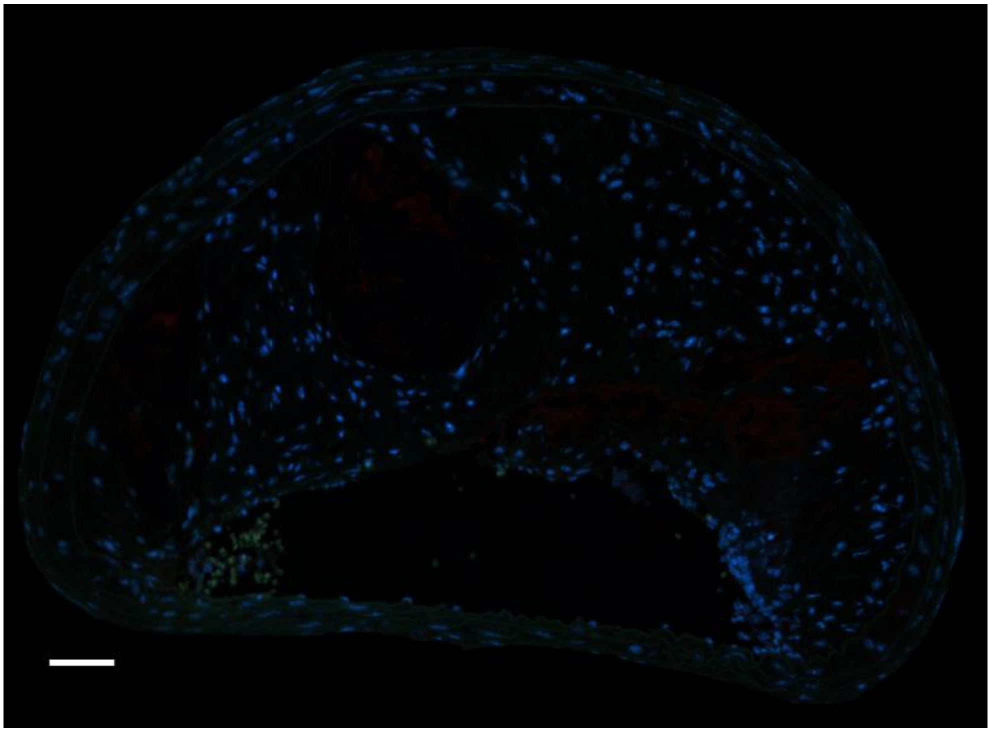

Supplementary Figure 3. Negative control for immunofluorescence assays in BCA (scale bar: 50 µm). The image is from mice GAa775.

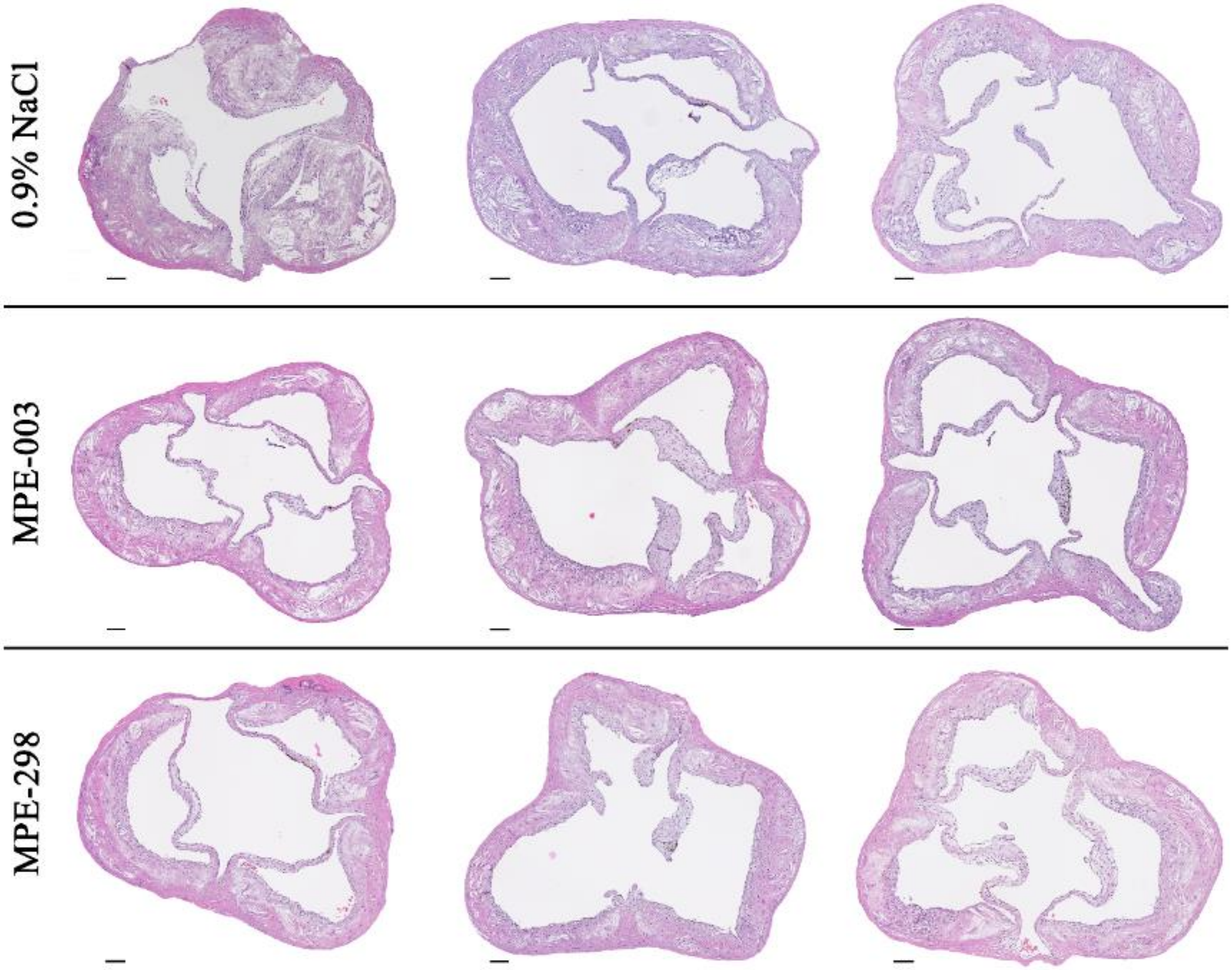

Supplementary Figure 4. Representative photomicrographs of aortic sinuses after staining with hematoxylin-eosin (scale bar: 100 µm). The photomicrographs of the first column are shown in Figure 1.

(A)

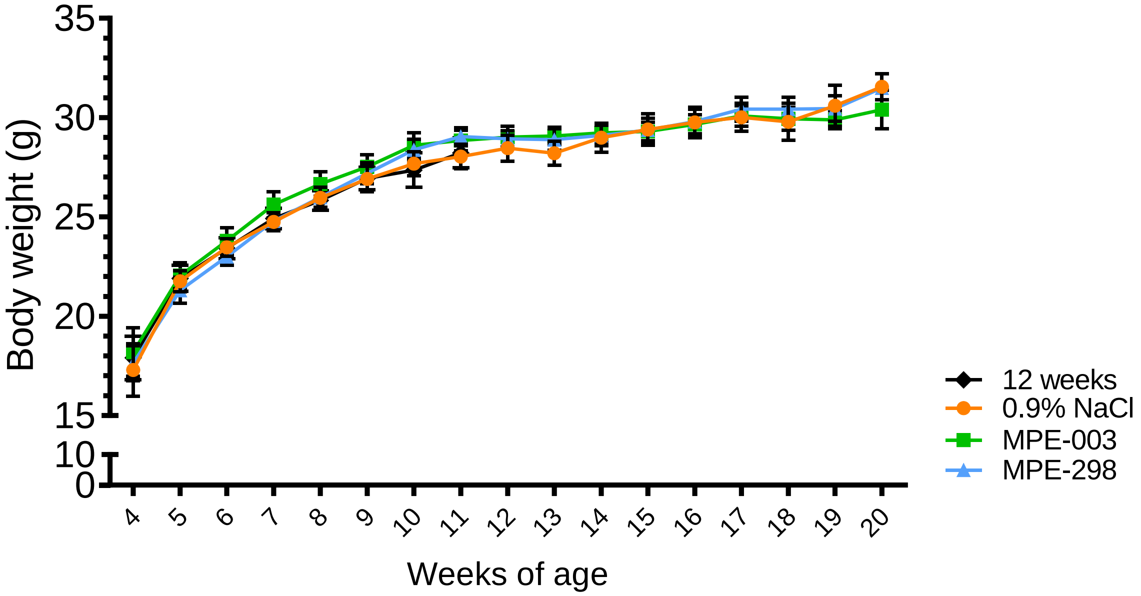

(B)

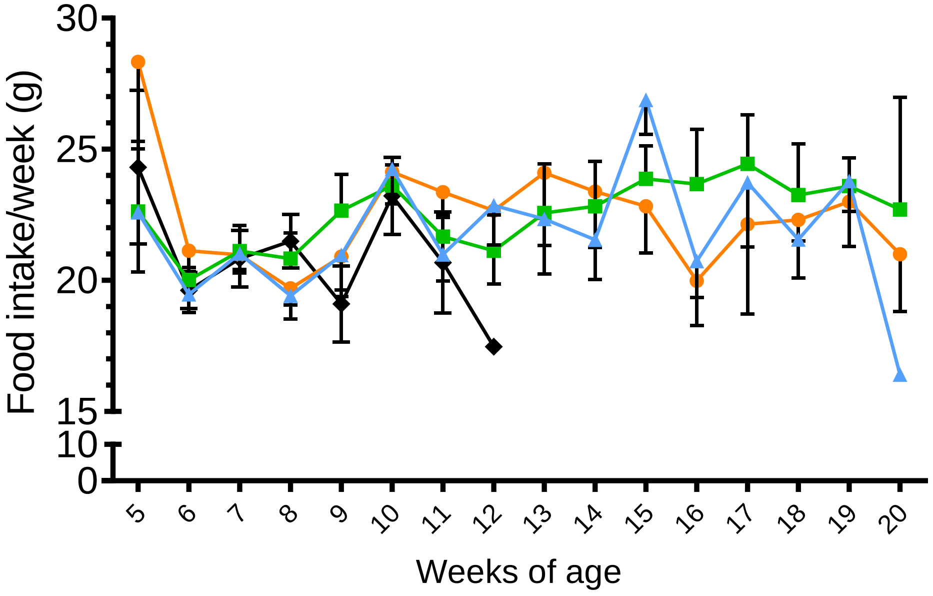

Supplementary Figure 5. Azapeptides did not affect body weight and food intake. ApoE-/- mice (A) mean body weight and (B) mean food intake per week. Azapeptides treatment was initiated at 12 weeks of age, until euthanasia at 20 weeks of age.

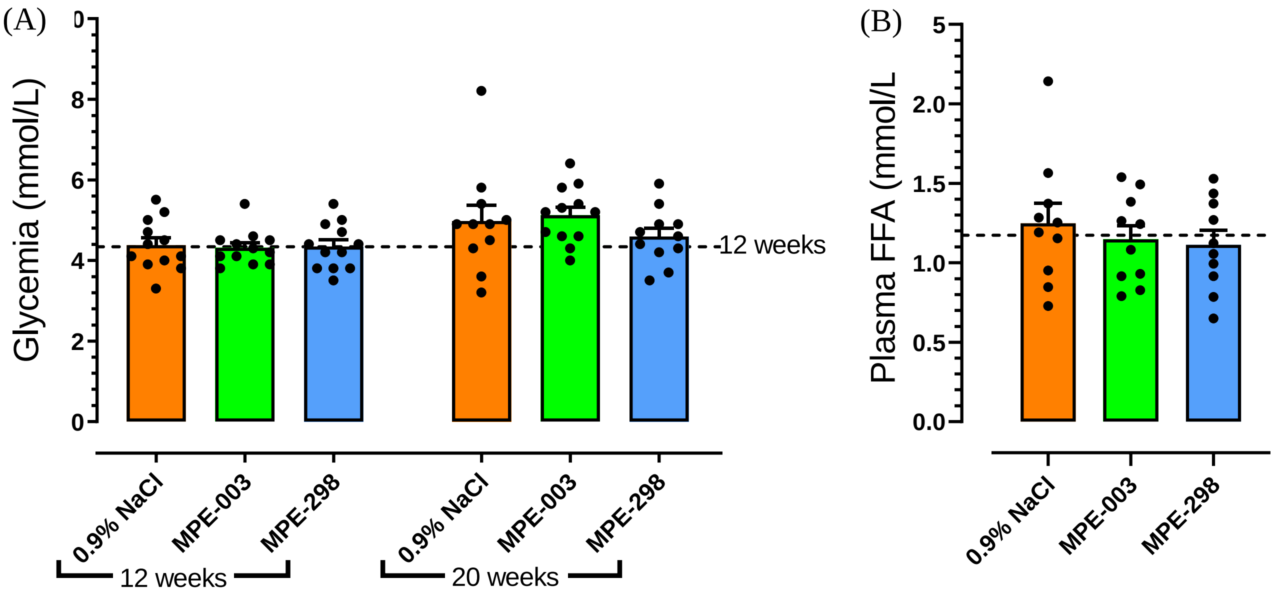

Supplementary Figure 6. Azapeptides did not affect (A) glycemia or (B) plasma free fatty acids (FFA) in apoE-null mice fed a HFHC diet for 16 weeks (4 to 20 weeks of age).

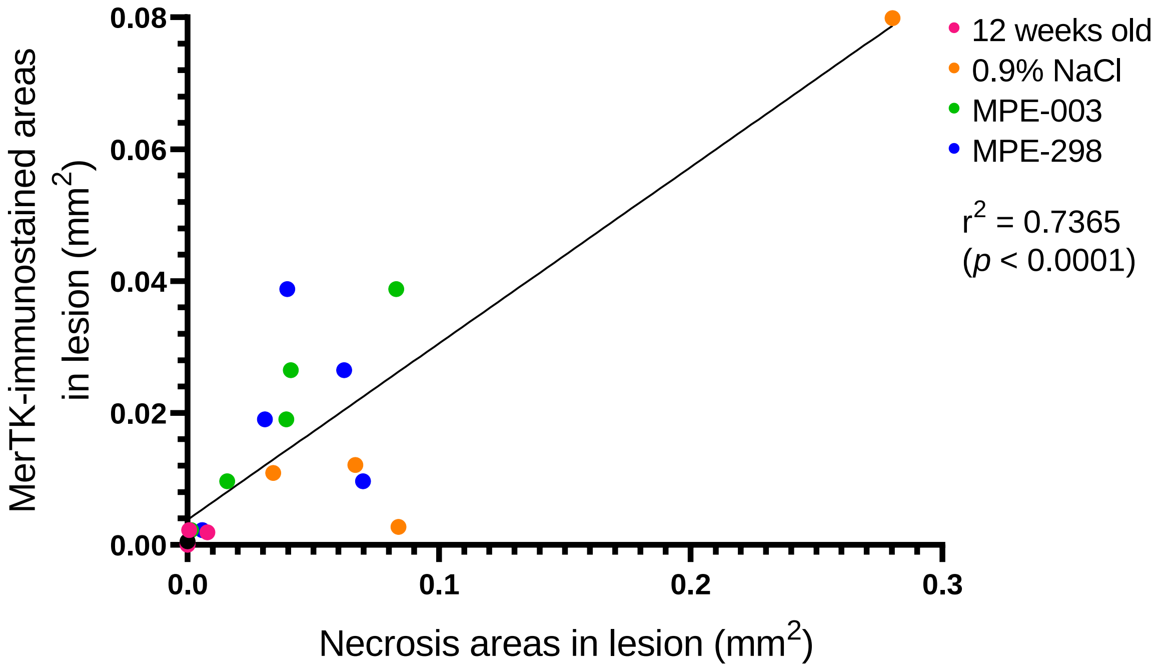

Supplementary Figure 7. Correlation between MerTK-stained areas and necrosis areas in BCA lesions. Each pink dot represents 12 weeks old mice, orange dot represents vehicle-treated mice, green dot represents MPE-003-treated mice and blue dot represents MPE-298-treated mice.
